# Supplementary material for: WordCommentsAnalyzer: A windows software tool for qualitative research
Source: F1000Res. 2018 Sep 4;7:536. Originally published 2018 May 3. [Version 2] doi: 10.12688/f1000research.14819.2 (PMC6137411; doi:10.12688/f1000research.14819.2)
Supplement: Supplementary file 3 [file f1000research-7-17614-s0002.tgz › b2a79496-4ada-47c4-9682-a99ce867836d.zip!/Eight Iranian Health Journals/Journal of Knowledge and Health_22.docx]

© Journal of Knowledge and Health. This work is licensed under CC BY 4.0 (https://creativecommons.org/licenses/by/4.0/).

Volume & Issue: دوره 7، شماره 4: 1391

Title: تأثير فعاليت بدني بر سطح سرمي لپتين و هورمون‌هاي توليدمثلي زنان

http://knh.shmu.ac.ir/index.php/site/article/view/68

مقدمه: هدف از مطالعه حاضر بررسی ارتباط بین تغییرات سطوح سرمی لپتین و هورمون‌های تولیدمثلی، متعاقب یک جلسه فعالیت بدنی در زنان فعال مي‌باشد. مواد و روش‌ها: 20 زن سالم، جوان و فعال تحت مطالعه قرار گرفتند. پس از 12 ساعت ناشتایی (در ساعت 8 صبح)، نمونه‌های خون پیش‌آزمون جمع‌آوری شد. سپس آزمودنی‌ها پروتکل نوارگردان بالک (به‌عنوان فعالیت بدنی منتخب) را به‌اجرا‌درآورده و بلافاصله بعد از آن نمونه خون پس‌آزمون گرفته‌شد. از آزمون t همبسته جهت مقایسه میانگین‌های پیش‌آزمون و پس‌آزمون و از ضریب همبستگی پیرسون برای بررسی روابط استفاده شد. سطح معناداری 05/0>P در‌نظر‌گرفته ‌شد. نتایج: غلظت هورمون‌های لپتین، LH و FSH متعاقب فعالیت بدنی تغییر معناداری نسبت‌به اندازه‌های پیش‌آزمون نداشتند (05/0<P)، اما غلظت هورمون‌های تستوسترون و استرادیول به‌طور معناداری افزایش ‌یافته ‌است (به‌ترتیب 023/0=P و 011/0=P). غلظت هورمون‌های تستوسترون و استرادیول با غلظت لپتین سرم همبستگی مستقیم و معناداري را نشان مي‌دهند (به‌ترتیب 47/0=r و 43/0=r)، اما رابطه معناداری بین غلظت LH یا FSH با غلظت لپتین سرم مشاهده نگردید (به‌ترتیب 21/0=r و 17/0=r). همچنين همبستگي معناداری بین تغییرات تستوسترون، استرادیول، LH و FSH با تغییرات غلظت هورمون لپتین وجود‌ نداشت. نتیجه‌گیری: غلظت تستوسترون و استرادیول با غلظت لپتین سرم رابطه مستقیم دارند، اما رابطه‌ای بین غلظت LH و FSH با غلظت لپتین سرم مشاهده نشد. همچنین به‌نظر‌می‌رسد بین تغییرات سطوح سرمی لپتین و هورمون‌های تولیدمثلی، متعاقب یک جلسه فعالیت بدنی در زنان فعال رابطه‌ای وجود‌ ندارد.

Title: بررسي اثربخشي درمان عقلانی، هیجانی و رفتاری به‌شيوه گروهي در کاهش استرس بیماران مبتلا به دیابت

http://knh.shmu.ac.ir/index.php/site/article/view/73

مقدمه: درمان عقلانی، هیجانی و رفتاری (Rational, Emotive Behavior Therapy) یکی از رویکردهای مبتنی‌بر بازسازی شناختی در درمان استرس می‌باشد. هدف پژوهش حاضر بررسی اثربخشی این درمان در کاهش استرس بیماران مبتلا به دیابت است. مواد و روش‌ها: در اين مطالعه مداخله‌اي، ابتدا 200 نفر از بيماران ديابتي مراجعه‌كننده به انجمن ديابت شهرستان كرج از‌نظر ميزان استرس بررسي شدند. سپس 30 نفر از بیمارانی كه نمرات استرس بالايي داشتند انتخاب و به‌صورت تصادفي به دو گروه آزمون و کنترل (هر گروه 15 نفر) تقسیم شدند. براي گروه آزمون، 8 جلسه آموزشی- درمانی 5/1 ساعته به‌صورت هفته‌ای یک‌بار برگزار گردید. جهت سنجش استرس از مقیاس نشانگان استرس (SSI) استفاده شد. تجزیه‌و‌تحلیل داده‌ها با استفاده از تحلیل کوواریانس انجام شد. نتایج: یافته‌های پژوهش حاکی از وجود تفاوت معنادار بین میانگین‌ گروه آزمایش و کنترل در تمام نشانگان استرس شامل جسمانی، هیجانی، شناختی و رفتاری بود. نتیجه‌گیری: نتایج پژوهش حاضر دال بر اثر‌بخش‌بودن REBT در کاهش استرس بیماران مبتلا به دیابت بوده‌ است. باتوجه به افزایش استرس بیماران دیابتی و مؤثر‌بودن مداخله روانی، توجه جدی به درمان‌های روانشناختی در این گروه بیماران، ضروری به‌نظر مي‌رسد.

Title: تأثیر یک ماه روزه‌داری و فعالیت بدنی منظم بر شاخص‌های هماتولوژی و بيوشيميايي خون

http://knh.shmu.ac.ir/index.php/site/article/view/71

مقدمه: روزه‌داری در ماه رمضان یک تکلیف مذهبی و باور اعتقادی است که بر همه مسلمانان سالم و بالغ واجب است. هدف از مطالعه حاضر، مقایسه‌ تأثیر یک ماه روزه‌داری و فعالیت ورزشی منظم بر شاخص‌های بیوشیمیایی و هماتولوژی خون مردان فعال و غیرفعال مي‌باشد. مواد و روش‌ها: در این مطالعه 26 مرد سالم 18 تا 30 سال در دو گروه روزه‌دار غیرفعال (13 نفر) و گروه روزه‌دار فعال (13 نفر) قرار گرفتند. از هر دو گروه دو نمونه خون جهت آزمايش‌هاي بيوشيميايي و هماتولوژي در روز اول و روز 29 ماه رمضان گرفته شد. داده‌ها با استفاده از آزمون آناليز واريانس تكراري تجزيه‌و‌تحليل شدند. نتایج: در هر دوگروه میانگین شاخص‌های هماتوکریت، تعداد گلبول‌های قرمز، TC، LDL، HDL، LDL/HDL، TC/HDL و VLDL در آخر ماه رمضان کاهش معناداری نسبت‌به ابتداي ماه رمضان داشته‌است. مقادیر HDL طی روزه‌داری در گروه روزه‌دار فعال (023/0P=) و گروه روزه‌دار غیرفعال (042/0P=) در ابتدا و انتهاي مطالعه افزایش معناداری یافته‌ است. کاهش سطوح FBS تنها در گروه روزه‌دار فعال معنادار بود (05/0>P). نتیجه‌گیری: روزه‌داری در ماه مبارک رمضان توأم با انجام فعالیت‌های ورزشی منظم می‌تواند تغييرات مثبتی در شاخص‌های هماتولوژیکی- بیوشیمیایی سرمی به‌وجود‌آورد که این می‌تواند به‌علت تغییر در رژیم غذایی و واکنش‌های زیست‌حیاتی بدن روزه‌داران به گرسنگی و فعالیت بدنی در طول ماه رمضان باشد.

Title: راهبردهاي كنترل فكر و بازداري پاسخ در افراد مبتلا به اختلال استرس حاد و افراد بهنجار

http://knh.shmu.ac.ir/index.php/site/article/view/74

مقدمه: اختلال استرس حاد با نشانه‌های تجزیه‌ای، تجربه مجدد، اجتناب و برانگیختگی بیش‌ازحد مشخص می‌شود. هدف پژوهش حاضر مقايسه راهبردهاي كنترل فكر و بازداري پاسخ در بيماران مبتلا به اختلال استرس حاد و افراد بهنجار است. مواد و روش‌ها: این پژوهش یک مطالعه مقايسه‌اي است. نمونه این پژوهش 40 بيمار مبتلا به اختلال استرس حاد و 40 فرد بهنجار ساكن در استان مازندران بودند که با روش نمونه‌گیری در دسترس انتخاب شدند. برای جمع‌آوری داده‌ها از مصاحبه تشخيصي جامع بين‌المللي، پرسش‌نامه‌ راهبردهاي كنترل فكر، آزمون استروپ و مقياس ضربه رويداد- تجديد‌نظر شده استفاده شد. نتايج: نتایج تحلیل واریانس چندمتغیری نشان داد که ميانگين نمرات راهبردهاي كنترل فكر (7/94) و بازداري پاسخ (6/12) در افراد مبتلا به اختلال استرس حاد، از افراد بهنجار (5/50 و 8/3) به‌طور معناداري بيشتر است. نتایج تحلیل رگرسیون چندگانه نشان داد که راهبردهاي كنترل فكر و بازداري پاسخ 75% از واریانس علائم اختلال استرس حاد را در افراد مبتلا به اين اختلال به‌طور معناداری تبیین می‌کنند. نتيجه‌گيري: اين یافته‌ها بیان‌ می‌کنند كه بیماران مبتلا به استرس حاد، راهبردهای کنترل فکر ناکارآمد‌تر و بازداری پاسخ بیشتری دارند. همچنین این یافته‌ها تلویحات مهمی در‌زمینه پيشگيري، آسیب‌شناسی روانی و درمان این بیماران دارند.

Title: بررسی تأثیر اجرای برنامه توان‌بخشی مبتنی‌بر جامعه بر وضعیت زندگی معلولان در ایران

http://knh.shmu.ac.ir/index.php/site/article/view/75

مقدمه: توان‌بخشی مبتنی‌بر جامعه، یک استراتژی جهت بهبود کیفیت زندگی معلولان می‌باشد. مطالعه حاضر با هدف تعیین تأثیر اجرای برنامه توان‌بخشی مبتنی‌بر جامعه، بر وضعیت زندگی معلولان ایران انجام شده‌است. مواد و روش‌ها: پژوهش حاضر كه در سال 1390 انجام شده، از نوع مطالعات مقايسه‌اي است. روش نمونه‌گیری به‌صورت چندمرحله‌ای بود. در مرحله اول، از بین استان‌های کشور 30% به‌روش نمونه‌گیری تصادفی و در مرحله بعد 50 شهرستان از بین 100 شهرستانی که مجری طرح توان‌بخشی مبتنی‌بر جامعه در این 10 استان بودند، انتخاب گردیدند. سپس در هر شهرستان 30 معلول به‌صورت تصادفی انتخاب و به‌طور‌کلی برای 1503 معلول، فرم جمع‌آوری اطلاعات تیزون تکمیل گردید. براساس اين پرسش‌نامه، توانايي‌هاي فرد معلول قبل و بعد از اجراي برنامه توان‌بخشي مبتني‌بر جامعه سنجيده شد. تجزيه‌و‌تحليل داده‌ها با استفاده از آزمون‌ آماري ويلكاكسون انجام‌شد. نتایج: ميانگين (±انحراف معیار) سني افراد شرکت‌کننده در مطالعه 2/14±30 سال بود. اکثر واحدهای پژوهش، مجرد و مرد بودند. اغلب افراد (6/36%)، معلولین جسمی و حرکتی بودند و مهم‌ترین علت معلولیت، نقایص مادرزادی بود. پس از اجرای برنامه توان‌بخشی، امتیاز توانایی‌های روزمره زندگی، توانایی‌های حرکتی، ارتباطات اجتماعی، توانایی‌های شغلی، کیفیت زندگی، مبنع درآمد، وضعیت استخدام، میزان مشارکت در زندگی اجتماعی و وضعیت آموزش، به‌طور معناداری ارتقا یافته‌بود. در تمامی حیطه‌ها بین اجرای برنامه توان‌بخشي مبتني‌بر جامعه و افزایش توانایی‌های معلولین، ارتباط معناداری مشاهده شد (001/0P<). نتیجه‌گیری: اجرای توان‌بخشی مبتنی‌بر جامعه تأثیر مثبتی بر بهبود وضعیت زندگی معلولین داشته است.

Title: بررسی شیوع و عوامل مرتبط با رتینوپاتی دیابتی در شاهرود

http://knh.shmu.ac.ir/index.php/site/article/view/76

مقدمه: رتینوپاتی دیابتی، یکی از عوارض خطرناک دیابت می‌باشد که می‌تواند منجر‌به نابینایی گردد. هدف از اين مطالعه، تعيين شيوع و عوامل مرتبط با رتينوپاتي ديابتي در مراجعان به واحد ديابت شاهرود مي‌باشد. مواد و روش‌ها: براي 625 نفر از مراجعين به مركز دیابت شاهرود از سال 1378 لغایت 1388، معاينه كامل عمومی و تخصصی چشم‌پزشکی انجام ‌شد. نتایج: میانگین سنی واحدهای پژوهش 7/11± 9/47 سال بود. میانگین مدت‌زمان ابتلا به دیابت 8/53± 5/48 ماه بود. در فوندوسکوپی انجام‌شده 442 نفر (7/70%) وضعيت طبیعی و 183 نفر (3/29%) رتینوپاتی دیابتی داشتند که 4/23% از نوع غیر‌پرولیفراتیو و 9/5% از نوع پرولیفراتیو بود. رابطه معناداری بین رتینوپاتی دیابتی با سن بیمار (005/0P<)، دیابت نوع دو (003/0P<)، مدت‌زمان ابتلا به دیابت (003/0P<) و مقدار قند خون ناشتا (008/0P<)  وجود داشت، ولی رابطه آماری معناداری بین جنس (621/0=P) و نوع داروی ضدّقند مصرفي (586/0=P) با رتینوپاتی دیابتی یافت ‌نشد. نتیجه‌گیری: با تشخیص زودرس دیابت و کنترل عوامل تسریع‌کننده ابتلا به رتینوپاتی دیابتی و درمان‌های مؤثر و به‌موقع، مي‌توان پيشرفت بيماري را در اين افرد کاهش ‌داد.

Title: مقايسه هزينه خدمات ارائه‌شده در مراكز بهداشتي- درماني شهري و روستايي شهرستان شاهرود

http://knh.shmu.ac.ir/index.php/site/article/view/78

مقدمه: تحلیل هزینه‌ها زمینه استفاده بهینه از منابع را فراهم‌نموده، سبب بهبود عملکرد نظام سلامت خواهد شد. مطالعه حاضر به‌منظور مقایسه هزينه خدمات ارائه‌شده‌ در مراكز بهداشتي- درماني شهري و روستايي شهرستان شاهرود انجام‌ شده ‌است. مواد و روش‌ها: در اين مطالعه توصیفی- مقطعی کلیه مراکز بهداشتی- درمانی شهری و روستایی شهرستان شاهرود در سال 1388بررسي شدند. داده‌های موردنياز با بیش از 70 فرم گردآوري شدند. براي توصیف داده‌ها از نرم‌افزار تحلیل هزینه، Excel و SPSS استفاده گردید. نتايج: از كل 496 نفر پرسنل بهداشتي- درماني، 5/42% در مراكز شهري و 5/57% در مراكز روستايي مشغول به‌ کار بودند. در سطح کلیه مراکز شهری و روستایی 1/66% هزینه‌ها مربوط‌به هزینه‌های پرسنلی و 9/33% مربوط‌به سایر هزینه‌ها بود. تعداد خدمات ارائه‌شده در مراکز شهری 777383 و در مراکز روستایی 828888 بود. میانگین هزینه یک خدمت در مراکز شهری 27228 ریال و در مراکز روستایی 34354 ریال، میانگین هزینه یک پوشش در مراکز بهداشتی- درمانی شهری 29601 و در مراکز روستایی 37266 ريال محاسبه گرديد. درمجموع تعداد خدمات و پوشش‌های ارائه‌شده در مراکز شهری کمتر از مراکز روستایی بود. نتيجه‌گيري: مهم‌ترین عامل تأثیرگذار بر هزینه تمام‌شده خدمات هزینه‌های پرسنلی بوده که تعدیل و استفاده بهینه از نیروها، استفاده از فناوري‌هاي جدید و استفاده از توان‌بخش خصوصی در ارائه خدمات در مراکز شهری می‌تواند در بهره‌وری بهتر از منابع نقش مؤثری ایفا نماید.
